# Supplementary material for: Metabolomes of mitochondrial diseases and inclusion body myositis patients: treatment targets and biomarkers
Source: EMBO Mol Med. 2018 Oct 29;10(12):e9091. doi: 10.15252/emmm.201809091 (PMC6284386; doi:10.15252/emmm.201809091)
Supplement: Supplementary file 1 — Expanded View Figures PDF [file EMMM-10-e9091-s001.pdf]

Expanded View Figures

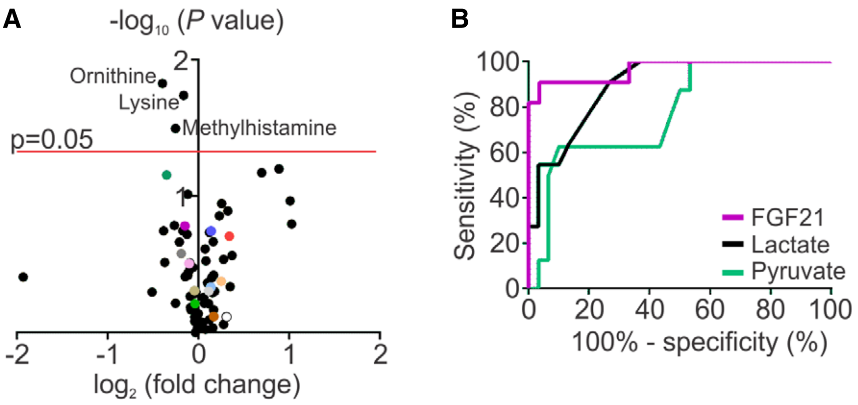

Figure EV1. Volcano plot of blood metabolites and ROC curves for reported / conventional blood biomarkers.

A Volcano plot of all the blood metabolites of male (n = 14) and female (n = 16) controls. Colours in the volcano plot indicate the same most relevant and/or significantly changed metabolites as in Figs 1 and 2.

B ROC curves for conventional biomarkers in blood of muscle-manifesting PEO and MELAS/MIDD patients (n = 11) compared to controls (n = 30).

Data information: AUC of FGF21 0.97 (95% CI: 0.91–1.03, $P < 0.0001$), lactate 0.897 (95% CI: 0.8–0.99, $P = 0.00012$) and pyruvate 0.78 (95% CI: 0.6–0.95, $P = 0.017$).

 © 2018 The Authors
