# Supplementary material for: Metabolomes of mitochondrial diseases and inclusion body myositis patients: treatment targets and biomarkers
Source: EMBO Mol Med. 2018 Oct 29;10(12):e9091. doi: 10.15252/emmm.201809091 (PMC6284386; doi:10.15252/emmm.201809091)
Supplement: Supplementary file 2 — Table EV1 [file EMMM-10-e9091-s002.docx]

**Table EV1:** Metabolites changed in mitochondrial patients with increased insulin resistance compared to normoglycemic patients

| **Metabolites** | **FC** | **Raw p-value** | **FDR** |
| --- | --- | --- | --- |
| AMP | 2.5 | 0.008 | 0.35096 |
| Glyceraldehyde | -1.4 | 0.009 | 0.35096 |
| Ribose-5-P | 2.4 | 0.0144 | 0.37573 |
| Glutathione reduced | -2.4 | 0.0216 | 0.42107 |
| Phenylalanine | -1.5 | 0.0412 | 0.55405 |
| Tryptophan | -1.2 | 0.0426 | 0.55405 |
