# Supplementary material for: Metabolomes of mitochondrial diseases and inclusion body myositis patients: treatment targets and biomarkers
Source: EMBO Mol Med. 2018 Oct 29;10(12):e9091. doi: 10.15252/emmm.201809091 (PMC6284386; doi:10.15252/emmm.201809091)
Supplement: Supplementary file 3 — Table EV2 [file EMMM-10-e9091-s003.docx]

**Table EV2:** Cross-validation of PLS-DA model with LOOCV method

|  |  | **Number of components** | | | | |
| --- | --- | --- | --- | --- | --- | --- |
| **Disease group** | **CV** | **1** | **2** | **3** | **4** | **5** |
| **IOSCA blood** | Accuracy | 0.8667 | 1* | 0.9333 | 0.9333 | 0.9333 |
|  | R2 | 0.8642 | 0.9597 | 0.9894 | 0.9984 | 0.9998* |
|  | Q2 | 0.4948 | 0.5818 | 0.6313 | 0.6328 | 0.6468* |
| **MIRAS blood** | Accuracy | 0.7436 | 0.7692 | 0.7949 | 0.8205* | 0.7949 |
|  | R2 | 0.5354 | 0.711 | 0.8393 | 0.9212 | 0.9567* |
|  | Q2 | 0.1684 | 1.1507 | 0.1317 | 0.146 | 0.2025* |
| **PEO blood** | Accuracy | 0.8889 | 0.8889 | 0.8889 | 0.9167* | 0.8889 |
|  | R2 | 0.535 | 0.7972 | 0.8873 | 0.9166 | 0.9527* |
|  | Q2 | 0.2844 | 0.3007 | 0.3229 | 0.3315* | 0.1858 |
| **MELAS blood** | Accuracy | 0.8857 | 0.9143* | 0.9143 | 0.9143 | 0.8857 |
|  | R2 | 0.5534 | 0.7179 | 0.838 | 0.9065 | 0.9421* |
|  | Q2 | 0.2911 | 0.3236* | 0.2379 | 0.12 | 0.0265 |
| **IBM blood** | Accuracy | 1* | 1 | 1 | 1 | 1 |
|  | R2 | 0.8793 | 0.9834 | 0.9934 | 0.999 | 0.9996* |
|  | Q2 | 0.7159 | 0.8089 | 0.8551* | 0.8531 | 0.8527 |
| **NMD blood** | Accuracy | 0.68 | 0.72 | 0.8 | 0.8 | 0.84* |
|  | R2 | 0.5404 | 0.7595 | 0.9254 | 0.9665 | 0.9872* |
|  | Q2 | 0.2696 | 0.3084 | 0.3384 | 0.3664 | 0.3768* |
| **MIRAS carriers blood** | Accuracy | 0.7826* | 0.7174 | 0.6739 | 0.6087 | 0.587 |
|  | R2 | 0.4745 | 0.6459 | 0.7171 | 0.8184 | 0.8513* |
|  | Q2 | 0.3124* | 0.2494 | 0.024 | -0.6184 | -1.0133 |
| **MIRAS muscle** | Accuracy | 0.6* | 0.5333 | 0.6 | 0.6 | 0.5333 |
|  | R2 | 0.609 | 0.8497 | 0.9357 | 0.9661 | 0.9871* |
|  | Q2 | -0.2443 | -0.2359* | -0.2368 | -0.2921 | -0.3573 |
| **PEO muscle** | Accuracy | 0.6667 | 0.9167* | 0.8333 | 0.75 | 0.75 |
|  | R2 | 0.5683 | 0.87 | 0.9871 | 0.9972 | 0.9996* |
|  | Q2 | 0.1334 | 0.3037 | 0.3242* | 0.3203 | 0.3216 |

* marks the highest accuracy/ R2/ Q2 values with the lowest number of components in each group
